# Supplementary material for: An entropy-controlled objective chip for reflective confocal microscopy with subdiffraction-limit resolution
Source: Nat Commun. 2023 Sep 20;14:5838. doi: 10.1038/s41467-023-41605-y (PMC10511456; doi:10.1038/s41467-023-41605-y)
Supplement: Supplementary file 1 — Supplementary Information [file 41467_2023_41605_MOESM1_ESM.pdf]

Supplementary Materials for

An entropy-controlled objective chip for reflective confocal microscopy with subdiffraction-limit resolution

Jun He[1,#], Dong Zhao[1,#], Hong Liu[2], Jinghua Teng[2,*], Cheng-Wei Qiu[3,*], Kun Huang[1,*]

[1]Department of Optics and Optical Engineering, University of Science and Technology of China, Hefei, Anhui 230026, China

[2]Institute of Materials Research and Engineering, Agency for Science Technology and Research (A*STAR), 2 Fusionopolis Way, #08-03, Innovis, Singapore 138634, Singapore

[3]Department of Electrical and Computer Engineering, National University of Singapore, 4 Engineering Drive 3, Singapore 117576, Singapore

[#] *J. H. and D. Z.* contributed equally to this work.

[*]Corresponding authors: K. H. (huangk17@ustc.edu.cn), J. T. (jh-teng@imre.a-star.edu.sg) or C. Q. (chengwei.qiu@nus.edu.sg)

Table of Contents

Section 1. Mathematical basis for Strehl ratio and focal size of an objective chip 2

Section 2. Optimization of the objective chip 5

Section 3. Experimental characterization of focusing properties of the objective chip 10

Section 4. Measuring the focusing efficiency of the objective chip 11

Section 5. Measuring modulation transfer function (MTF) of the objective chip 12

Section 6. Roles of collection objective in scanning confocal microscopy 13

Section 7. Simulated images by using different microscopies for comparison 15

Section 1. Mathematical basis for Strehl ratio and focal size of an objective chip

To reveal the relationship between the information entropy S and optical properties of an objective chip, we investigate its Strehl ratio and focal size under different deviation probability p_1. For a given p_1, the relative Strehl ratio and focal size change because the locations of the deviated zones in the objective chip are different. It means that the relative Strehl ratio and focal size have a certain range with the fixed minimum and maximum values, which can be determined mathematically by using diffraction properties of each zone.

First, we derive the minimum and maximum Strehl ratios. Because the binary-phase objective chip is reported here with the modulation phase of 0 and π, we can directly use its complex modulation of 1 and -1, respectively. In our design strategy, the objective chip is functionally divided into a binary-phase FZP and an N-ring phase mask. The phase of π in the N-ring phase mask realizes the reversal (from 1 to -1, or from -1 to 1) of the complex modulation. Assuming that the odd and even rings have the phase of 0 and π respectively, it means that the electric fields contributed by the zones in the even rings are removed from those of the standard FZP and then are used to interfere constructively with those of the zones in the odd rings. For the objective chip containing a N-ring phase mask, Eq. (3) describing the total electric fields of our objective chip can be rewritten as

$$E_{\text{chip}}(u,v,z) = \sum_{n=0}^{N-1}(-1)^n\left[\sum_{m=M_n}^{m=M_{n+1}}(-1)^m A_m\right] = \sum_{m=1}^{m=M}(-1)^m A_m - 2\times\sum_{m\in\boldsymbol{\mathcal{R}}}(-1)^m A_m$$

$$= E_{\text{FZP}}(u,v,z) - 2\times\sum\nolimits_{m\in\boldsymbol{\mathcal{R}}}(-1)^m A_m, \qquad \text{(S1)}$$

where $\boldsymbol{\mathcal{R}}$ denotes a set of the indices of all the phase-reversed zones (*i.e.*, contained in the even rings of the N-ring phase mask). For our design strategy used in this work, we can obtain the number of the set $\boldsymbol{\mathcal{R}}$ by using $M \cdot p_1$, where M is the total number of zones in the corresponding FZP and p_1 is the deviation probability (see Eq. (1) in the main text) of the phase-reversed zones. Considering its university, Eq. (S1) is valid for all binary-phase planar diffractive lenses.

After substituting Eq. (S1) into Eq. (2) of main text, the relative Strehl ratio can be expressed as

$$SR = \frac{I_{\text{chip}}(0,0,z=f)}{I_{\text{FZP}}(0,0,z=f)} = \frac{|E_{\text{chip}}(0,0,z=f)|^2}{|E_{\text{FZP}}(0,0,z=f)|^2} = \frac{\left|E_{\text{FZP}}(0,0,f)-2\times\sum_{m\in\boldsymbol{\mathcal{R}}}(-1)^m\cdot A_m(0,0,f)\right|^2}{|E_{\text{FZP}}(0,0,z=f)|^2}, \qquad \text{(S2)}$$

where $E_{\text{FZP}}(0, 0, f)$ is in phase with $a_0 = (-1)^m A_m(0,0,f)$. Meanwhile, for different m, the item a_0 is also in phase with each other and nearly a constant with a slow variation from $a_{0\min} = 0.87$

to $a_{0\max} = 2$, which can be numerically calculated by using the rigorous Rayleigh-Sommerfeld diffraction integral. Due to their feature of slow variation, the on-axis intensity a_0 from all the zones with the zone indices $m \in \mathcal{R}$ are assumed to be identical. Thus, $\sum_{m\in\mathcal{R}} a_0$ can be approximated by $a_0 p_1 M$. By applying these assumptions, Eq. (S2) can be approximated as

$$SR = (1 - 2a_0 p_1 M/\sqrt{I_{\mathrm{FZP}}})^2, \tag{S3}$$

where $E_{\mathrm{FZP}} = \sqrt{I_{\mathrm{FZP}}}$ and $0.87 \le a_0 \le 2$. Eq. (S3) reveals the direct link between SR and p_1(which is a key parameter to evaluate the disorder of a planar diffractive lens). Based on it, we can evaluate the range of SR by determining its minimum and maximum values. By carrying out straightforward derivations, we have the minimum value $SR_{\min} = \left(1 - 2a_{0\max} p_1 M/\sqrt{I_{\mathrm{FZP}}}\right)^2$ for $0 \le p_1 \le \sqrt{I_{\mathrm{FZP}}}/(2Ma_{0\max})$ and $SR_{\min} = 0$ for $\sqrt{I_{\mathrm{FZP}}}/(2Ma_{0\max}) \le p_1 \le 0.5$, and the maximum value $SR_{\max} = \left(1 - 2a_{0\min} p_1 M/\sqrt{I_{\mathrm{FZP}}}\right)^2$ for $0 \le p_1 \le \sqrt{I_{\mathrm{FZP}}}/(Ma_{0\max} + Ma_{0\min})$ and $SR_{\max} = \left(1 - 2a_{0\max} p_1 M/\sqrt{I_{\mathrm{FZP}}}\right)^2$ for $\sqrt{I_{\mathrm{FZP}}}/(Ma_{0\max} + Ma_{0\min}) \le p_1 \le 0.5$. Note that the analytical $SR_{\min}$and $SR_{\max}$ depend on only the deviation probability p_1, and are therefore valid for various objective chips with different N.

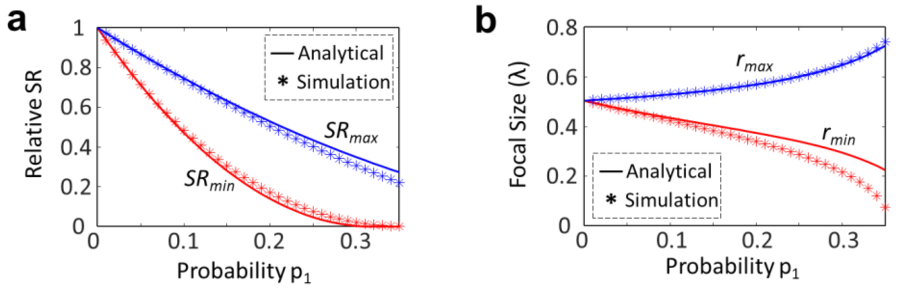

Supplementary Fig. 1. Analytical and simulated Strehl ratio (a) and focal size (b) under the different deviation probability p_1. The analytical Strehl ratios are determined by using Eq. (S3), which the analytical focal sizes are obtained by numerically solving Eqs. (S4) and (S5).

To verify the analytical $SR_{\min}$ and $SR_{\max}$, we have simulated the range of SR by using the proposed objective chip with a 5-ring phase mask. The limited $N = 5$ of the phase mask allows us to go through all possible solutions quickly without any optimization because all the A_{m} can be calculated ahead. By controlling the number and position of the phase-reversed zones in the 5-few ring phase mask, the simulations are implemented within the range of $0 \le p_1 \le 0.35$ with an interval of 0.01, which is enough here because low disorder p_1 is important to develop the

objective chip with good balance between imaging and super-focusing. The simulated $SR_{\min}$ and $SR_{\max}$ are shown in Supplementary Fig. S1a, exhibiting good agreement with their analytical values. Their slight deviations come from the approximations made during its derivation. Therefore, these results have confirmed that the analytical $SR_{\min}$ and $SR_{\max}$ give a good prediction for the range of Strehl ratio.

Second, the focal size of an objective chip with different p_1 can also be predicted by using Eq. (S1). As shown in Eq. (S1), the electric field of the objective chip is taken as the coherent super-position of the electric fields from all the zones. Diffraction behavior of each zone is important in predicting the focal size of the objective chip. Because the width of each zone is small, the diffraction field from each zone is mainly determined by its focusing angle between the outmost boundary of each zone and optical axis. When the focusing angle is large, the relative focal spot size of diffraction field from one zone is small; vice versa. For an objective chip, its maximum focal spot is achieved when the contribution from the outermost zones is small, where the electric field at the focal plane is

$$E_{\mathrm{chip}}(u,v,f) = E_{\mathrm{FZP}}(u,v,z) - 2 \times \sum_{m=M\cdot(1-p_1)}^{m=M} (-1)^m A_m, \tag{S4}$$

where the phases of the outermost zones are reversed with a deviation probability p_1. From Eq. (S4), we numerically predict the maximum focal size, which depends on only p_1. In contrast, when the phases of the inner zones are reversed, the minimum focal size can be predicted by using the electric field at the focal plane

$$E_{\mathrm{chip}}(u,v,f) = E_{\mathrm{FZP}}(u,v,z) - 2 \times \sum_{m=0}^{m=M\cdot p_1} (-1)^m A_m, \tag{S5}$$

By using Eqs. (S4) and (S5), we can calculate the maximum and minimum focal spots, as shown in the solid lines in Supplementary Fig. S1b. Because both Eqs. (S4) and (S5) have no limitation about the number *N*, we use our proposed objective chip with a 5-ring phase mask to verify the predicted minimum ($r_{\min}$) and maximum ($r_{\max}$) focal size. Similarly, we go through all the possible solutions by changing the number and position of the phase-reversed zone in the second and fourth rings of the few-ring phase mask, which can be implemented together with the above calculation of the Strehl ratio. The simulated $r_{\min}$ and $r_{\max}$ are provided in Supplementary Fig. 1b. Both the predicted and simulated $r_{\max}$ agree with each other for the interested range of $0 \leq p_1 \leq 0.35$. However, for the case of $r_{\min}$, the discrepancy between the prediction and simulation increases

with the increment of p_1, which is caused by the large error of the approximation in Eq. (S5). In fact, for a large p_1, the disorder in the objective chip is higher than the predicted one in Eq. (S5), so that the minimum focal spot has more choices with a larger range than that predicted by Eq. (S5). Despite this, our prediction in Eq. (S5) shows the same decreasing tendency when p_1 increases, thereby confirming the validity of the predicted focal sizes.

Section 2. Optimization of the objective chip

According to the design strategy described in the main text, we implement the optimization of the objective chip with four key steps, as discussed below.

2.1. Determining the radius of each belt in a standard zone plate

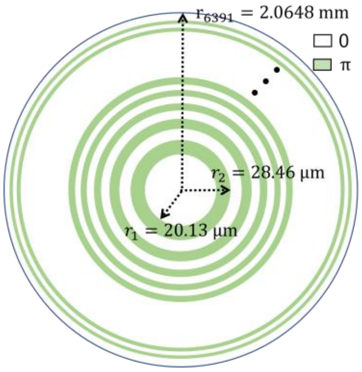

Supplementary Fig. 2. Sketch of standard zone plate with 6391 belts. The imaging parameters are $\lambda = 405$ nm, $f = 250$ μm, which indicates a radius of 2 mm.

Considering the non-paraxial feature of this objective chip, a rigorous formula of standard binary phase Fresnel zone plate (BPFZP) should be used to calculate the radius r_m of the mth belt with

$$r_m = \sqrt{(\frac{\lambda}{2}m)^2 + m\lambda f}, \tag{S6}$$

where $m = 0, 1, 2, \ldots, M$, the wavelength $\lambda = 405$ nm, the focal length $f = 1$ mm. In our design, the total number M of belts in this BPFZP is $M = 6391$ and the radius of BPFZP is ~2.0648 mm, which yields a numerical aperture (*NA*) of 0.9, as shown in Supplementary Fig. 2. The binary phase is employed to enhance optical efficiency of the objective chip.

2.2. Calculating the focal field of each belt in the BPFZP

Benefiting from our design strategy, no new ring is created during our optimization because all the radii can be described by Eq. (S6). It means that the focal field of light from each zone can be calculated ahead of optimization and then stored in a database, so that we directly revisit the

relative focal field during the optimization. Thus, the time cost will be significantly shorten. Since we need to evaluate the lateral focal size and the longitudinal depth of focus, both focal fields along the radial (*i.e.*, r) and longitudinal (*i.e.*, z) direction are calculated ahead, where the positons of interest are: 1) the lateral positions $0 \leq r \leq \lambda$ at the focal plane $z = 1000$ μm; 2) the longitudinal positions $950\ \mu m \leq z \leq 1050\ \mu m$ at the on-axis position $r = 0$. By using Rayleigh-Sommerfeld diffraction theory without any approximation for high accuracy, we calculate the focal fields of each belt along the lateral and longitudinal positions, and store them in two matrices (*i.e.*, A_r and A_z, see Supplementary Figs. 3a and 3b) respectively. According to the electric field stored in the 1th, 3000th and 6391th column of A_r and A_z, the normalized intensity of diffraction field at two target positions of corresponding belts of BPFZP are exemplified in Supplementary Figs. 3c and 3d, respectively. To show its convenience, the focal fields at two target positions for a BPFZP can be calculated as

$$E(r, z = f) = \sum_{m=0}^{m=M} (-1)^m A_m(r),$$
$$E(r = 0, z) = \sum_{m=0}^{m=M} (-1)^m A_m(z), \quad \text{(S7)}$$

where $A_m(r)$ is the radial-position electric field of light diffracting from the mth belt in the zone plate and saved in the mth column of A_r, $A_m(z)$ is the longitudinal-position electric field of light diffracting from the mth belt in the zone plate and stored in the mth column of A_z. According to this definition, $A_m(r) = A_m(z) = 0$=0 when $m = 0$. Based on these database, we can calculate any focal field of the objective chip only if the phase of each zone is given.

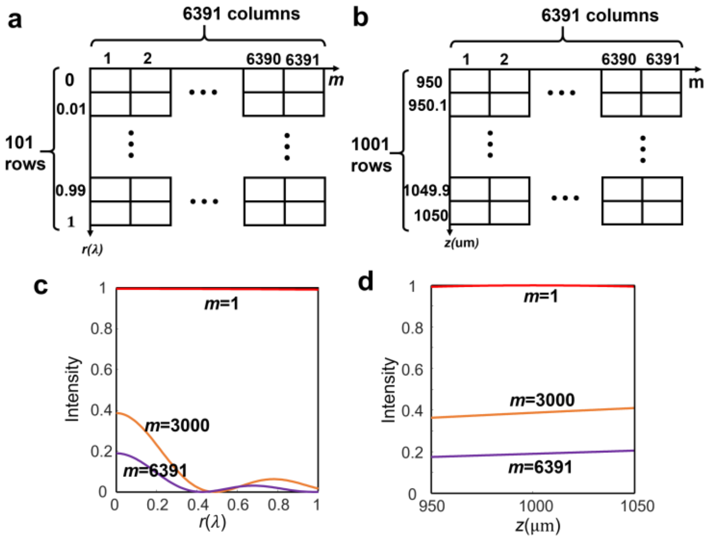

Supplementary Fig. 3. Data preparation for optimization. The A_r **(a)** and A_z **(b)** database show

the way of saving the pre-calculated data in our optimization. The normalized line intensity at the focal plane **(c)** and on the optical axis **(d)** of the 1th, 3000th and 6391th belt of BPFZP.

2.3. Optimizing the 5-ring phase mask

To optimize the detailed structures of the 5-ring phase mask, we use the well-matured particle swarm optimization (PSO) algorithms that have been used frequently to design various lenses, especially for super-oscillation and super-critical lenses. Considering the limited number of the few-ring mask, the optimization will be implemented with its standard version of the PSO algorithm. For a 5-ring mask, the dimension of particle D is 5. In our algorithm, the size or population of the particle is 20. The details and flowchart of PSO are shown in Supplementary Fig. 4. Since the electric fields (A_r and A_z) have been calculated in advance, the calculation of fitness of each particle in iteration can be finished quickly, as shown below.

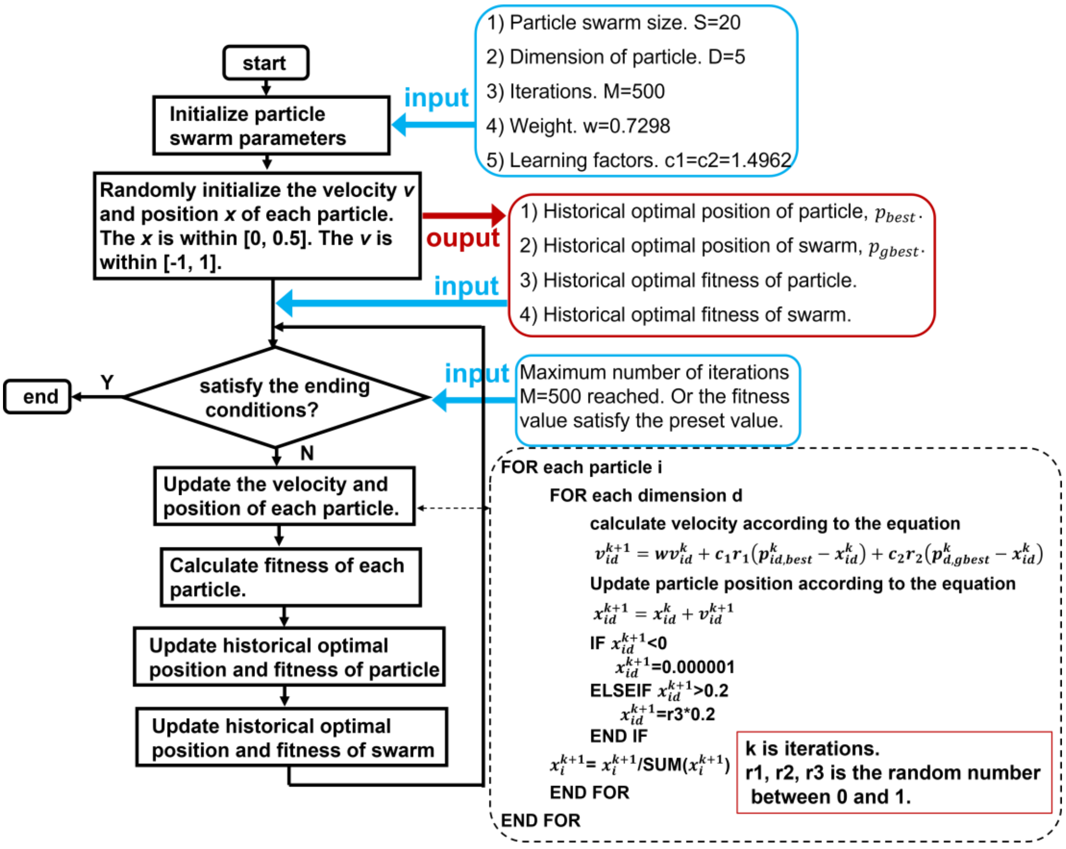

Supplementary Fig. 4. The detailed flowchart of our built PSO.

To correlate the particle parameters $[x_1, x_2, x_3, x_4, x_5]$ with the unknown structures of the designed objective chip, we define the relative *NA* at the each boundary of the 5-ring phase mask by using

$$NA_n = \frac{\sum_{i=1}^{n} x_i}{\sum_{i=1}^{5} x_i} \cdot NA_0, \tag{S8}$$

where *n* is the ring number of the few-ring phase mask, $NA_0 = 0.9$ is used in this work. From Eq. (S8), one can induce that$NA_5 = NA_0$, which means that the outer boundary of the 5-th ring in the phase mask refers to the maximum radius of 2.046 mm. The five parameters in each particle are related with the difference of *NA* between two neighboring rings. In this definition, we have built the one-to-one relationship between the particle parameters and the structures of the objective chip. In addition, such a definition will offer full degree of freedom to go through all the possible solutions because each x_i can be valued within $0 < x_i < \infty$. The normalization factor of $1/\sum_{i=1}^{5} x_i$ is quite helpful to make the maximum value of NA_0, thus enabling each particle to yield a physically meaningful objective chip. Thus, the universal properties of design an objective chip are maintained in our definition, which is an important step to implement this optimization. In one iteration, each x_i is updated with the PSO algorithm (as described in the dashed rectangle of Supplementary Fig. 4). Based on the optimized x_i, we derive the *NA* parameters in Eq. (S8), from which we find each R_n in the designed objective chip by using the equation $R_n/\sqrt{{R_n}^2 + f^2} = NA_n$ (or $R_n = NA_n \cdot f/\sqrt{1 - NA_n^2}$).

To avoid the creation of additional finer structures when combining the zone plate and 5-ring mask, the optimized R_n is approximated by the closet r_m, which is labelled as $r_{M_n} = \rho_n$. Thus, we can find all the M_n, hereby fixing the geometric structures of the few-ring phase mask in each iteration.

According to the updated M_n and the well-built database, we can calculate the electric fields of objective chip at two positions of interest:

$$E(r, z = f) = \sum_{n=0}^{n=4} \sum_{m=M_n}^{m=M_{n+1}+1} (-1)^{m+n} A_m(r),$$

$$E(r = 0, z) = \sum_{n=0}^{n=4} \sum_{m=M_n}^{m=M_{n+1}} (-1)^{m+n} A_m(z) \ , \tag{S9}$$

From Eq. (S9), we can obtain the relative intensity $I_r = |E(r, z = f)|^2$ and $I_z = |E(r = 0, z)|^2$. Then, two root-mean-square error (RMSE) between ideal and simulated patterns are calculated as

$$RMSE_1 = \sqrt{\frac{\left(I_r - I_r^{ideal}\right)^2}{N_r}} \ ,$$

$$RMSE_2 = \sqrt{\frac{(I_z - I_z^{ideal})^2}{N_z}}, \tag{S10}$$

where the ideal radial-position intensity I_r^{ideal}=$|J_0(krNA)|^2$, J_0 is the zero-order Bessel function of the first kind, $k = 2\pi/\lambda$ is wave vector, the radial position *r* is valued between 0 and λ with an interval of 0.01λ (*i.e.*, the sampling number is $N_r = 101$), the ideal longitudinal-position intensity $I_z^{ideal} = e^{-\frac{(z-f)^2}{DOF^2}}$, $DOF = \frac{\lambda}{1-\sqrt{cos\theta}}$, $sin\theta = NA_0 = 0.9$, the longitudinal position *z* is valued between 950 μm and 1050 μm with an sampling interval of 0. 1 μm (*i.e.*, the sampling number is $N_r = 1001$). The RMSE$_1$ and RMSE$_2$ are used to evaluate the electric fields at the radial and longitudinal positions. Based on them, we can build the cost function by using $CF = C \cdot RMSE1 + RMSE2$, where the positive constant *C* can be adjusted according to any special requirement. In this work, $C = 2$ is used to realize the sub-diffraction-limit focusing.

After 500 iterations in the PSO algorithm, we finally obtain the $M_1 = 283$, $M_2 = 850$, $M_3 = 1046$, $M_4 = 1258$ and $M_5 = 6391$, and the additional phase mask can be sketched in Supplementary Fig. 5. The combination between standard zone plate (Supplementary Fig. 2) and additional phase masks (Supplementary Fig. 5a) forms the objective chip. The simulated intensity profiles at the longitudinal and radial positions are provided in Figs. 2b and 2c of main text, respectively.

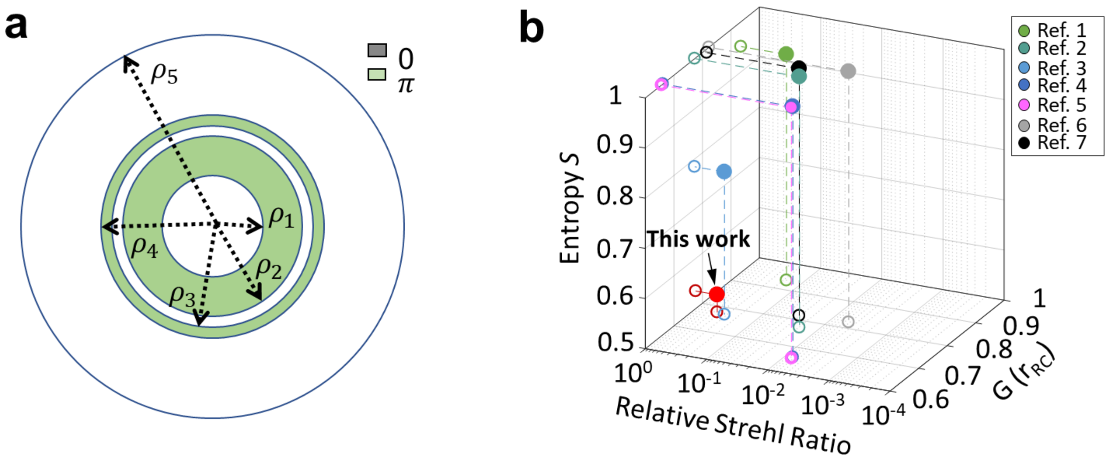

Supplementary Fig. 5. Design results of a 5-ring phase mask. (a) Phase profile of the optimized few-ring mask. The ideal phase difference $\Delta\varphi$ between two neighboring rings is π. **(b)** Entropy *S*, relative Strehl ratio and focal size of other reported planar diffractive lenses with the structural parameters (which is used to output p_1 for calculating entropy *S*) provided in their corresponding publications. This figure is an extension of Fig. 1d in the main text for a better observation due to

the overlay of data.

To show the difference from the previous planar diffractive lenses, we provide the entropy, relative Strehl ratio and focal size of various reported lenses[1-7] in Supplementary Fig. 5b, which is an extension of Fig. 1d in the main text for a better observation. From both figures, we can conclude that the entropy S of our proposed objective chip is the closet to the equilibrium point $S_0 = 0.5$, implying the good balance between imaging and super-focusing. Note that, although Ref. 3 has nearly identical focal size and relative Strehl ratio to ours, its entropy S is much higher than $S = 0.535$ in our objective chip, implying that its imaging capability is poor. For the other lenses, their entropy approaches 1, which is a natural result when a lens is designed only for super-focusing with high disorder. Therefore, the information entropy is a good measurement of evaluating the imaging and super-focusing capabilities in a straightforward way.

Section 3. Experimental characterization of focusing properties of the objective chip

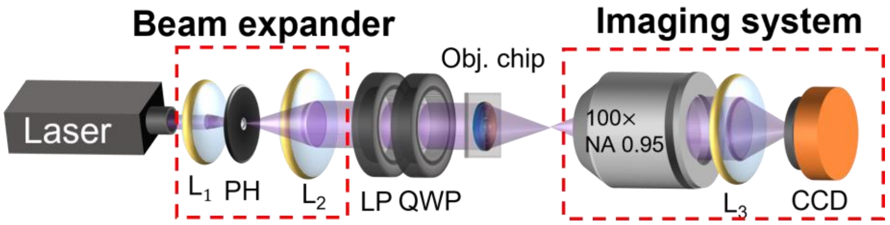

Supplementary Fig. 6. Experimental setup for characterizing the focusing capability of the fabricated objective chip. The beam expander consists of lenses L_1 and L_2 (with the focal lengths of 25.4 mm and 300 mm respectively) and a 25 μm-diameter pinhole. The imaging system consists of 100× objective with 0.95 NA, a lens L_3 (with a focal length 400 mm) and a CCD camera. LP: linear polarizer. QWP: quarter-wave plate; Obj. chip: objective chip;

To characterize the focusing capability of objective chip, we use the experimental setup as shown in Supplementary Fig. 6. The beam from a $\lambda = 405\,\text{nm}$ laser is reshaped by a beam expander (consisting of L_1, L_2, and a 25 μm-diameter pinhole), yielding a fundamental Gaussian beam with the diameter of ~1 mm. To generate circularly polarized illumination, a linear polarizer and quarter-wave plate are employed for obtaining the highly axisymmetric focal spot. Then, a sub-diffraction-limit focal spot generated by objective chip is projected by using the imaging system (composed of a 100× objective with 0.95 *NA* and a lens L_3) onto the CCD camera.

The focusing process of this objective chip is recorded dynamically in Supplementary Movie

1, which presents the focused spot near the focal plane. Such a movie shows the same intensity profiles that are similar to those in Fig. 2b of main text. All these experimental results have confirmed the good focusing with our proposed objective chip.

Section 4. Measuring the focusing efficiency of the objective chip

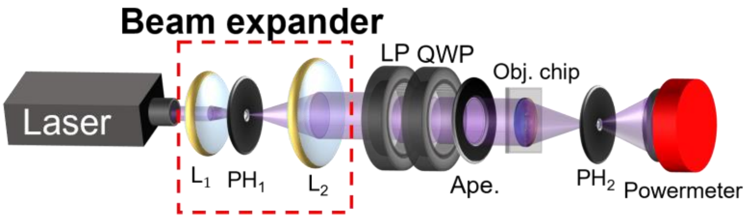

Supplementary Fig. 7. The schematic diagram of the experimental setup for measuring the focusing efficiency of the objective chip. The beam expander is composed of two lenses L_1 and L_2 (with their focal lengths of 25.4 mm and 300 mm, respectively) and a 25 μm-diameter pinhole (PH_1). LP: linear polarizer; QWP: quarter-wave plate; Ape.: aperture; Obj. chip: objective chip; PH_2: pinhole with 150 μm diameter.

The focusing efficiency of the objective chip is measured by using the experimental setup sketched in Supplementary Fig. 7. To obtain quasi-plane-wave illumination, we reshape a $\lambda = 405\,nm$ laser into a ~1 mm-diameter fundamental Gaussian beam by using a beam expander (containing L_1, L_2, and a 25 μm-diameter pinhole PH_1). A circular polarizer composed of a linear polarizer (LP) and a quarter-wave plate (QWP) is used to convert the polarization of the incident beam into the circular polarization, which is helpful to achieve the circular focal spot under the high-NA focusing condition. To remove the background light from high-diffraction-order rings diffracted by the pinhole PH_1, an iris aperture with its transmission area slight larger than the entrance of the objective chip is utilized here to keep the nearly uniform incident light. The second pinhole PH_2 with a 150 μm diameter is placed at focal plane of objective chip to select the focused power, which is recorded as I_1 by using a power meter. Due to the high NA of 0.9 in the objective chip, the divergence angle of light passed through PH_2 is large. Therefore, the power-meter is located close to the PH_2 for complete collection of all focused light. By removing the objective chip and the PH_2 simultaneously, we can measure the total power of the incident beam, as recorded as I_2. Finally, we obtain the experimental focusing efficiency of objective chip, *i.e.*, $\eta = \frac{I_1}{I_2} = 12.3\%$, which is tightly consistent with the theoretical 18.7%. Although such an experimental efficiency is not so high

compared with the dielectric metasurfaces, we will show its capability in optical imaging and focusing for high-quality confocal microscopy.

Section 5. Measuring modulation transfer function (MTF) of the objective chip

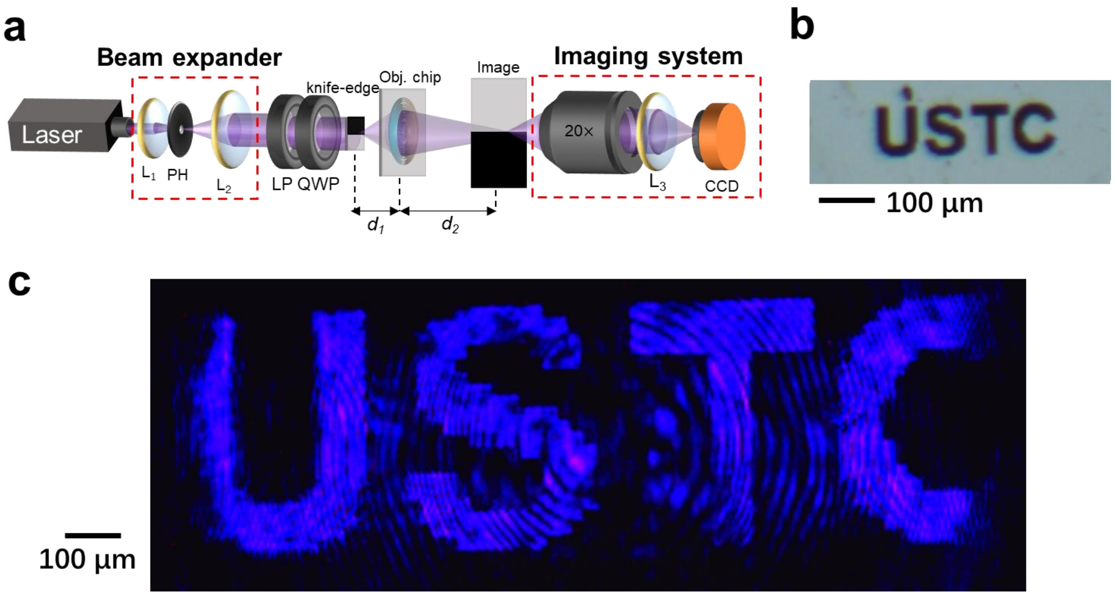

Supplementary Fig. 8. Sketch for the experimental setup to measure MTF of the objective chip when working in an imaging mode. **(a)** The beam expander is made of two lenses L_1 and L_2 (with their focal lengths of 25.4 mm and 300 mm respectively) and a 25 μm-diameter pinhole (PH). The imaging system consists of a 20× objective, a lens (with a focal length of 400 mm) and a CCD camera. LP: linear polarizer; QWP: quarter-wave plate; Obj. chip: objective chip; d_1 and d_2 are the object and image distances, respectively. **(b-c)** Experimental measurement of field of view by using a large object "USTC" **(b)** with its horizontal length of 310 μm. The relative image is shown in **(c)**.

To measure the MTF of the objective chip, a self-made setup presented in Supplementary Fig. 8 is implemented to demonstrate its imaging properties in a transmission mode for a better experimental operation. Note that, the configuration of transmission or reflection will not influence the imaging properties of the objective chip. Similarly, a fundamental Gaussian beam with circular polarization is obtained by using a beam expander and a circular polarizer, and then works as the illumination beam of a knife-edge object (a 140 nm-thick Cr film coated on quartz substrate), which is mounted on 3-dimensional piezo stage (PI). To directly image the knife-edge, the objective chip mounted on a mechanical stage is placed behind the knife-edge with its structure side closer to the knife-edge for the collection of the transmitted light. To adjust the object distance d_1, we move the

imaging system to see the knife-edge and the structure surface of the objective chip respectively and record their corresponding positions z_1 and z_2. Thus, the object distance can be evaluated roughly by using $d_1 = |z_1 - z_2|$. We tune the position of objective chip so that the object distance $d_1 = 1.2f$ ($f = 1$ mm is the focal length of objective chip) is achieved, which yielding the relative imaging distance of $d_2 = 6f$ (see Fig. 2f in the main text). The captured image is shown in the insert of Fig. 2f in the main text. To show its imaging process of the knife edge, we provide a dynamic movie (see Supplementary Movie 2) that records the out-of-focus and in-focus images by moving the axial position of knife-edge near $d_1 = 1.2f$ and simultaneously fixing the position of the imaging system. These experimental results clearly show the good imaging ability of our proposed objective chip.

To test its field of view, we use a larger object of "USTC" that has a horizontal length of 310 μm, see Supplementary Fig. 8b. The object "USTC" is placed at $z = 1.2f$, which means its magnification of 5X. The resulting image is shown in Supplementary Fig. 8c, which shows the clear image with the slightly blurred horizontal edges. It means that a bigger object with its dimension larger than 310 μm cannot be imaged with clear edges. This indicates that the field of view is 310 μm×310 μm at the magnification of 5X. These results show the wide-field image capability of our developed objective chip.

Section 6. Roles of collection objective in scanning confocal microscopy

To highlight the importance of the collection objective in a scanning confocal microscopy, we implement the numerical simulation of the imaging processes by using the theory of scanning confocal microscopy [8]. When the scanned object is not an infinitesimal point, the imaging resolution of scanning confocal microscopy is determined by the *NA* of collection objective. In our simulations, the theoretically focused spot by using our objective chip is taken as the focal field h_1 of the condenser lens while the PSF of collection objective is the well-known Airy spot $h_2 = \frac{J_1(krNA)}{krNA}$, where *NA* is the numerical aperture the collection objective, $k = 2\pi/\lambda$ is the wave number, λ is the wavelength and r is the radial coordinate. The transmission T of the nano-objects is taken as its original pattern without considering the light-structure interaction, for the simplicity of the entire simulation process. For a certain scanning position (x_s, y_s), the electric field at the pinhole plane (*i.e.*, the imaging plane of the collection objective) can be written as $(h_1 \cdot T) \otimes h_2$, where $\otimes$ stands for

the convolution operation. After selected by the pinhole with a circular aperture, the total power is taken as the value of image at this scanning position (x_s, y_s). Thus, if all the positions in the object are scanned, we can achieve the final image. All the codes are built in a Matlab software. Supplementary Fig. 8 show the simulated images by using collection objectives with different *NA*s that changes from 0.5 to 0.9. From these simulated results, one can see that, when $NA \leq 0.6$, the scanning images will be distorted seriously with a bad imaging resolution. In comparison, the imaging resolution is high if $NA \geq 0.7$, which suggests the smallest *NA* of 0.7 to achieve high-resolution imaging in a SCM. Therefore, the *NA* of collection objective is also critical for high-quality imaging of real objects in SCM. Moreover, the resolution reduces with the increment of slit width, because the contrast of image is getting lower. This result is consistent with the fact that the fixed 200nm-CTC-distance double slits will not be resolved when the double silt merges into one slit with the continuing increment of slit width.

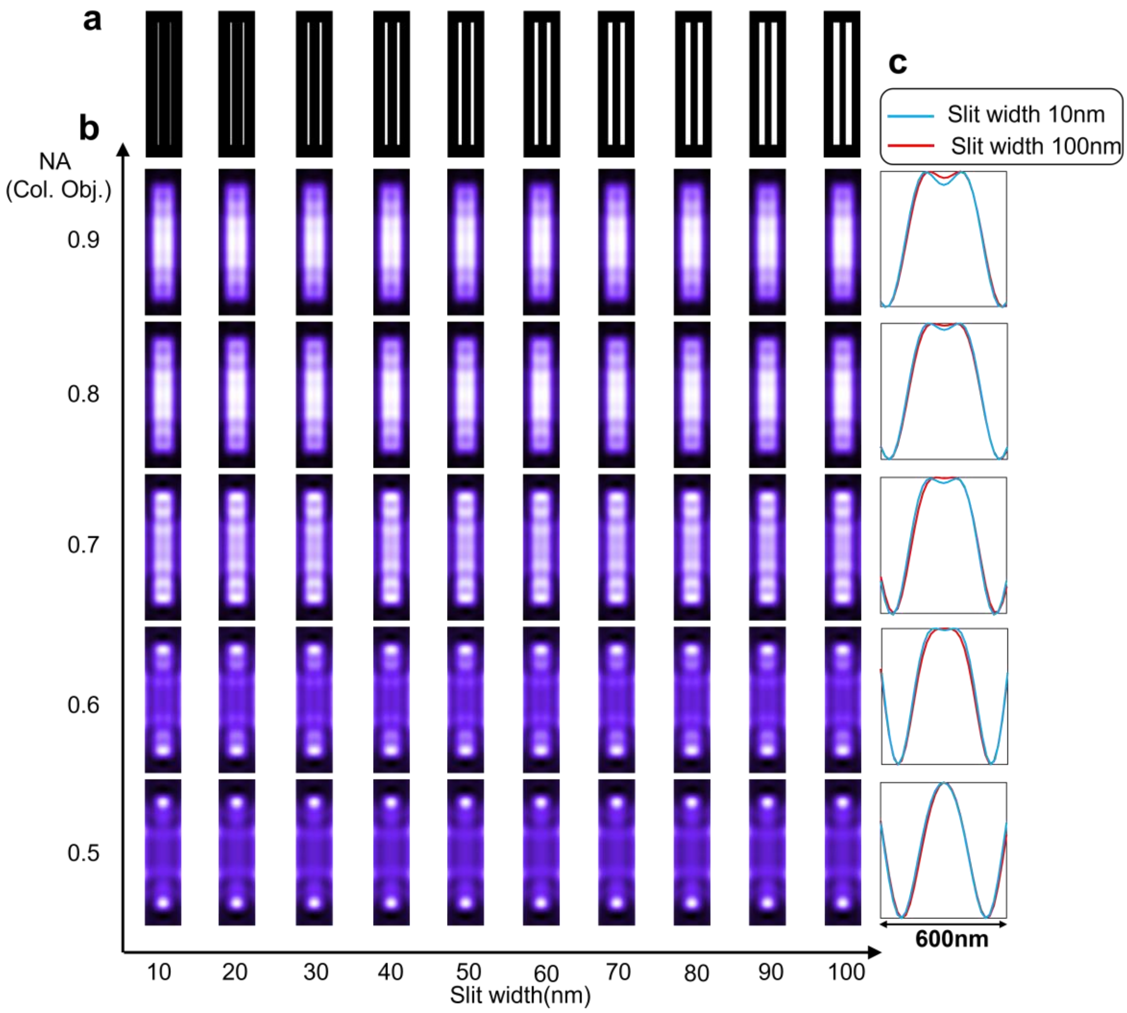

Supplementary Fig. 9. Simulated images of double slits by using collection objectives with

different *NA*s. (a) Double slits with the center-to-center distances of 200 nm and the silt widths ranging from 10nm to 100nm. The length of slits are 2 μm. **(b)** Simulated scanning images under different *NA*s (0.5-0.9) and slit widths (10nm-100nm). **(c)** The line-scanning profiles of these slit images for the collection objectives with different *NA*s.

Section 7. Simulated images by using different microscopies for comparison

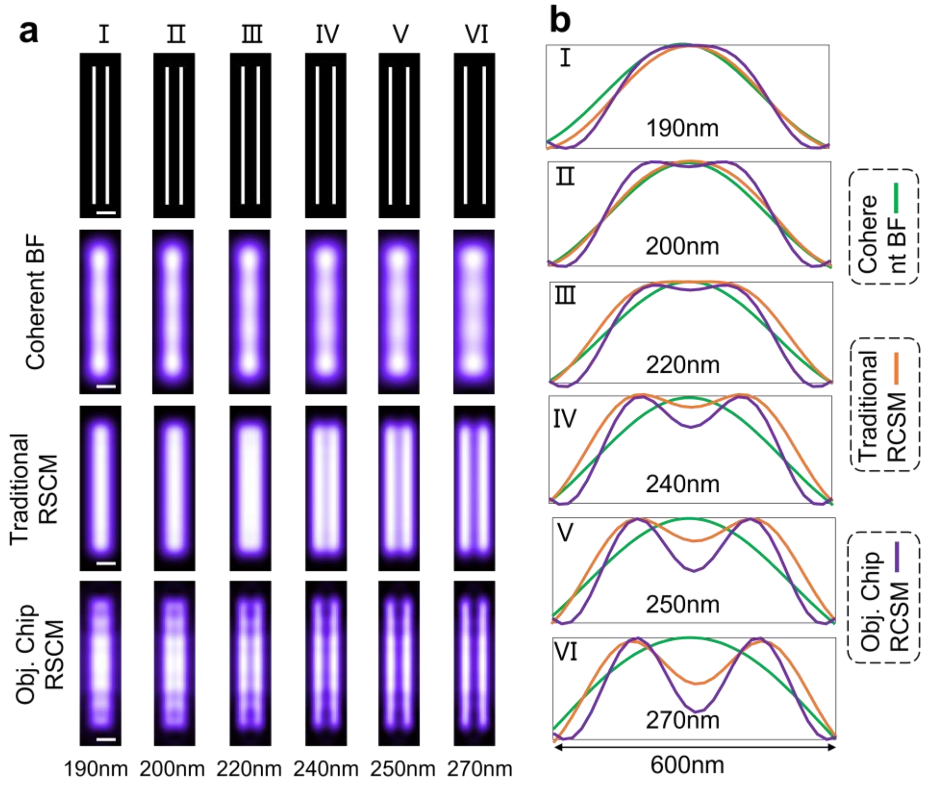

Supplementary Fig. 10. Simulated images of double slits with different CTC distances. (a) Sketch of double slits (top row) and their simulated images by using coherent bright field microscope (second row), traditional RSCM (third row) and objective-chip-based RSCM (forth row). The height and width of slit are 2 μm and 50 nm, respectively. The CTC distances of double slits are 190 nm, 200 nm, 220 nm, 240 nm, 250 nm and 270 nm, respectively. Image Size: 2.4 μm×0.6 μm. Scale bars: 300 nm. **(b)** The line-scanning profiles of these simulated images by addressing the CTC distances of double slits.

By using the imaging theory, we provide a numerical simulation of images by using different microscopies. To simulate the imaging results from different types of microscopes, these double slits with the fixed 50-nm width and varied CTC distance are employed as the objects for keep the consistence with our experimental cases. To simulate the images of doubles slits by coherent bright-

field microscope, the convolution between the objects and an Airy spot profile $\frac{J_1(krNA)}{krNA}$ with 0.9 NA is employed. Similarly, the simulation of traditional RSCM and objective-chip-based RSCM is implemented according to the theory of SCM, as mentioned in Ref [8]. Both PSFs of condenser lens and collector lens in the traditional RSCM are the Airy spots with the amplitude of $\frac{J_1(krNA)}{krNA}$, where $NA = 0.9$ for a fair comparison. For our objective-chip-based RSCM, the PSF of condenser lens is the simulated electric field of our objective chip and the PSF of collector lens is Airy spot $\frac{J_1(krNA)}{krNA}$ with a measured NA of 0.83.

Supplementary Fig. 10 shows the simulated images that reveal the different resolution in various microscopies. As observed in our experiment, the coherent bright-field microscopy cannot resolve any double slit, suggesting its low resolving power. But, the traditional SCM by using refraction-based objective can only resolve the double slits with the CTC distance large than 240 nm, which agrees with our experimental results in Fig. 3 of main text. To show the advantages of our proposed SCMs, we give a detailed comparison with the previously reported SCMs by presenting their various parameters. One can find that we have proposed the first planar-diffractive -lens-based reflective SCM and the ultra-long working distance at the level of millimeter. More importantly, we can use industrial DUV lithography to fabricate all these objective chips in a mass-product and low-cost way, which pushes this objective chip towards practical applications. Supplementary Fig. 11 shows the images of our fabricated objective chips, which validates the feasibility for industrial mass production.

Supplementary Table 1. A comparison among far-field label-free SCMs based on planar lenses

Reference	Material	Thickness of lens	Type of lens	λ	Ambient Medium	Working Mode	Working Distance	Substrate	Center-to-center Resolution
Rogers et al.[9]	Al	100 nm	Amplitude	640 nm	oil	Transmission	10.3 μm	quartz	315 nm(0.492λ)
Qin et al.[10]	Cr	100 nm	Amplitude	405 nm	air	Transmission	55 μm	quartz	228 nm(0.563λ)
Chen et al.[11]	TiO_2	600 nm	Phase	532 nm	oil	Transmission	125 μm	quartz	400 nm(0.752λ)
Yuan et al.[12]	Gold	100 nm	Amplitude	800 nm	air	Transmission	10 μm	quartz	320 nm(0.4λ)
Wang et al.[13]	MoS_2	10 nm	Amplitude	450 nm	air	Transmission	20 μm	sapphire	200 nm(0.444λ)
This work	SiO_2	527 nm	Phase	405 nm	air	Reflection	1000 μm	quartz	200 nm(0.494λ)

Note: The image magnifications of all these SCMs in Supplementary Table 1 are 1×.

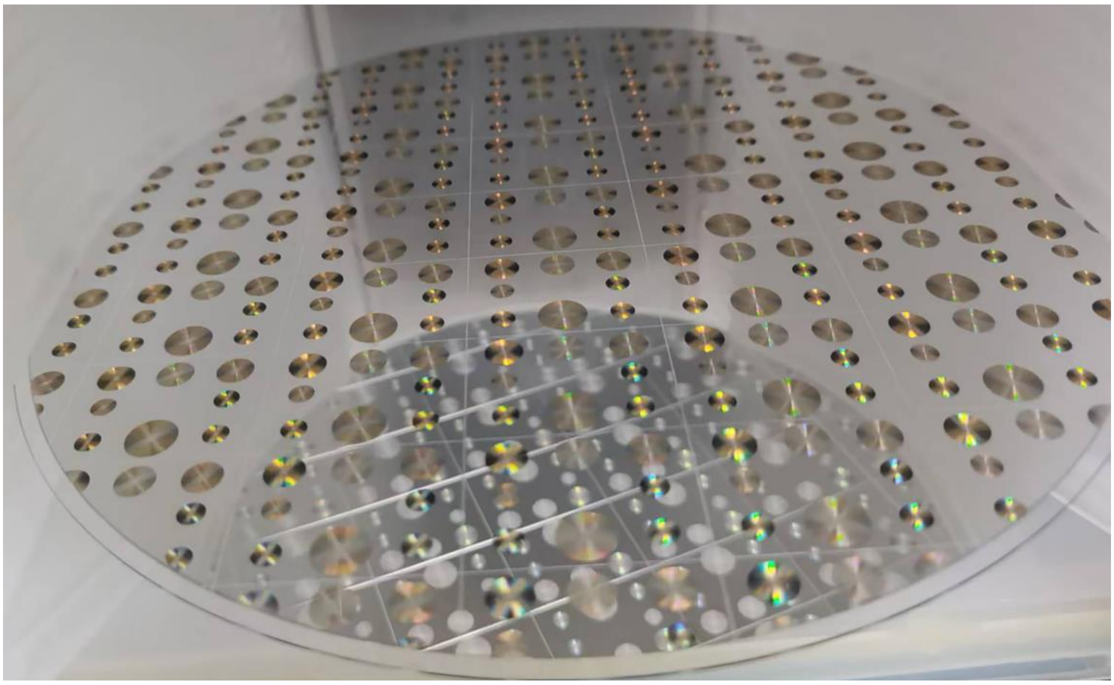

Supplementary Figure 11. Fabricated objective chips on an 8-inch quartz wafer by using DUV lithography

Supplementary References

[1]. Li, M., Li, W., Li, H., Zhu, Y., & Yu, Y., Controllable design of super-oscillatory lenses with multiple sub-diffraction-limit foci, *Sci. Rep.* **7**, 1(2017).

[2]. Diao, J., Yuan, W., Yu, Y., Zhu, Y., & Wu, Y., Controllable design of super-oscillatory planar lenses for sub-diffraction-limit optical needles, *Opt. Express* **24**, 1924(2016).

[3]. Wan, X., Shen, B., & Menon, R., Diffractive lens design for optimized focusing, *J. Opt. Soc. Am. A* **31**, B27(2014).

[4]. Liu, T., Liu, J., Zhang, H., & Tan, J., Efficient optimization of super-oscillatory lens and transfer function analysis in confocal scanning microscopy, *Opt. Commun.* **319**, 31(2014).

[5]. Liu, T., Shen, T., Yang, S., & Jiang, Z., Subwavelength focusing by binary multi-annular plates: design theory and experiment, *J. Opt.* **17**, 035610(2015).

[6]. Zhang, Z., Li, Z., Lei, J., Wu, J., Zhang, K., Wang, S., Cao, Y., Qin, F., & Li, X., Environmentally robust immersion supercritical lens with an invariable sub-diffraction-limited focal spot, *Opt. Lett.* **46**, 2296(2021).

[7]. Fang, W., Lei, J., Zhang, P., Qin, F., Jiang, M., Zhu, X., Hu, D., Cao, Y., & Li, X., Multilevel phase supercritical lens fabricated by synergistic optical lithography, *Nanophotonics* **9**, 1469(2020).

[8]. Huang, K., Qin, F., Liu, H., Ye, H., Qiu, C. W., Hong, M., Luk'yanchuk, B., & Teng, J., Planar diffractive lenses: fundamentals, functionalities, and applications, *Advanced Materials* **30**, 1704556(2018).

[9]. Rogers, E. T., Lindberg, J., Roy, T., Savo, S., Chad, J. E., Dennis, M. R., & Zheludev, N. I., A super-oscillatory lens optical microscope for subwavelength imaging, *Nat Mater* **11**, 432(2012).

[10]. Qin, F., Huang, K., Wu, J., Teng, J., Qiu, C. W., & Hong, M., A supercritical lens optical label-free microscopy: sub-diffraction resolution and ultra-long working distance, *Advanced Materials* **29**, 1602721(2017).

[11]. Chen, W. T., Zhu, A. Y., Khorasaninejad, M., Shi, Z., Sanjeev, V., & Capasso, F., Immersion Meta-Lenses at Visible Wavelengths for Nanoscale Imaging, *Nano Lett* **17**, 3188(2017).
[12]. Yuan, G., Rogers, K. S., Rogers, E. T., & Zheludev, N. I., Far-field superoscillatory metamaterial superlens, *Physical Review Applied* **11**, 064016(2019).
[13]. Wang, Z., Yuan, G., Yang, M., Chai, J., Steve Wu, Q. Y., Wang, T., Sebek, M., Wang, D., Wang, L., Wang, S., Chi, D., Adamo, G., Soci, C., Sun, H., Huang, K., & Teng, J., Exciton-Enabled Meta-Optics in Two-Dimensional Transition Metal Dichalcogenides, *Nano Letters*, 7964(2020).
