# Supplementary material for: An entropy-controlled objective chip for reflective confocal microscopy with subdiffraction-limit resolution
Source: Nat Commun. 2023 Sep 20;14:5838. doi: 10.1038/s41467-023-41605-y (PMC10511456; doi:10.1038/s41467-023-41605-y)
Supplement: Supplementary file 3 — Description of Additional Supplementary Files [file 41467_2023_41605_MOESM3_ESM.pdf]

Description of Additional Supplementary Files:

Supplementary Movie 1: Focusing of light near the focal plane of our objective chip. It was recorded by moving the collection system (containing a 0.95NA objective lens, a tube lens and a CCD camera) with a high-precision nano-stage.

Supplementary Movie 2: Imaging process of a “corner” object by using our objective chip. This video records the dynamic images when the “corner” object is moved across the ideal objective distance of z=1.2f.

Supplementary Movie 3: A video recording the reflective signal in our proposed reflective confocal microscopy when the sample is near the focal plane. The four-lobe intensity is the background signal that comes from the undesired reflection of light at the bare substrate of our objective chip.
